# Inhibition of Pathogenic Bacteria and Fungi by Natural Phenoxazinone from Octopus Ommochrome Pigments

**DOI:** 10.4014/jmb.2206.06043

**Published:** 2022-07-21

**Authors:** Lidianys María Lewis-Luján, Ema Carina Rosas-Burgos, Josafat Marina Ezquerra-Brauer, María Guadalupe Burboa-Zazueta, Simon Bernard Iloki Assanga, Teresa del Castillo-Castro, Giselle Penton, Maribel Plascencia-Jatomea

**Affiliations:** 1 Laboratorio de Microbiología y Micotoxinas, Departamento de Investigación y Posgrado en Alimentos, Universidad de Sonora, Blvd. Luis Encinas y Rosales S/N, Col. Centro, 83000 Hermosillo, Sonora, Mexico; 2 Departamento de Investigaciones Científicas y Tecnológicas, Blvd. Luis Encinas y Rosales S/N, Col. Centro, 83000 Hermosillo, Sonora, México; 3 Department of Biological Chemical Sciences. Sonora University, Blvd. Luis Encinas y Rosales. Col. Centro, 83000 Hermosillo, Sonora, México; 4 Department of Research on Polymers and Materials, Sonora University. Blvd. Luis Encinas y Rosales. Col. Centro, 83000 Hermosillo, Sonora, México; 5 Centro de Ingeniería Genética y Biotecnología, Ave 31 entre 158 y 190, Cubanacán, Playa, Habana, CP 6162, Cuba

**Keywords:** Ommochromes, xanthommatin, *Octopus vulgaris*, antioxidant, antimicrobial activity, fluorescence

## Abstract

Cephalopods, in particular octopus (*Octopus vulgaris*), have the ability to alter their appearance or body pattern by showing a wide range of camouflage by virtue of their chromatophores, which contain nanostructured granules of ommochrome pigments. Recently, the antioxidant and antimicrobial activities of ommochromes have become of great interest; therefore, in this study, the pH-dependent redox effect of the extraction solvent on the antioxidant potential and the structural characterization of the pigments were evaluated. Cell viability was determined by the microdilution method in broth by turbidity, MTT, resazurin, as well as fluorescence microscopy kit assays. A Live/Dead Double Staining Kit and an ROS Kit were used to elucidate the possible inhibitory mechanisms of ommochromes against bacterial and fungal strains. The results obtained revealed that the redox state alters the color changes of the ommochromes and is dependent on the pH in the extraction solvent. Natural phenoxazinone (ommochromes) is moderately toxic to the pathogens *Staphylococcus aureus*, *Bacillus subtilis*, *Salmonella* Typhimurium and *Candida albicans*, while the species *Pseudomonas aeruginosa* and *Pseudomonas fluorescens*, and the filamentous fungi *Aspergillus parasiticus*, *Alternaria* spp. and *Fusarium verticillioides*, were tolerant to these pigments. UV/visible spectral scanning and Fourier- transform infrared spectroscopy (FTIR) suggest the presence of reduced ommatin in methanol/ HCl extract with high intrinsic fluorescence.

## Introduction

The marine environment comprises complex ecosystems, and many organisms are known to possess bioactive compounds that function to provide self-defense or protection. Today, in addition to being of economic interest, cephalopods are important biological models with pertinence to food preservation and health [1, 2]. The octopus group makes up about a third of the global cephalopod population, with around 300 species found in waters around the world [3, 4].

The octopus has the ability to dynamically alter its appearance or body pattern by quickly displaying a wide range of camouflage and coloration to blend into its environment and escape detection by predators. It does so mainly by virtue of its chromatophores, which are neuromuscular organs, innervated directly from the brain, that contain nanostructured granules of ommochrome pigments. Chromophore pigments are a class of polycyclic aromatic tryptophan metabolites comprising phenoxazone derivatives substituted with the maximum characteristic of visible ultraviolet light (UV-vis) at 360 and 480 nm. They are the main products of tryptophan's metabolic oxidation pathway and among them are kinurenine, kinurenic acid, 3-hydroxyikinurenine, xanthommatin, and ommatin D. Three main families are composed of ommatin (low-molecular-weight, thermolabile and light-colored), ommidin, and ommin (high- molecular-weight, thermosensible, and related to intense colorations. The structure of the ommochromes is classified by a phenoxazone (ommatin) or phenotiazine (ommins and ommidins) ring. Structural changes of ommatins are based on ring substitutions (OR): dihydroxantommatin (R = H reduced form), rhodommatin (R = β-glucosyl-), ommatin D (R = SO_3_H), decarboxylated xanthommatin (COOH removed). The chemical structure offers an electronic delocalization system built on a polycyclic and asymmetric aromatic ring, which is composed of heteroatoms (N and O or S) [5-7]. The main functions performed by ommochromes are induction of skin color change, light filtration, antioxidant protection, and control of the spectral sensitivity of photoreceptors in reticular cells; they also function in detoxification of excess tryptophan [8-11].

Interest in finding antioxidants and antimicrobials from natural sources of animal origin has drawn attention to the cephalopod ommochrome pigments, owing to their ability to transfer hydrogens and electron atoms from the donor substituents of their phenoxazinone/phenothiazine rings. In this regard, studies have been carried out demonstrating that ommochromes can inhibit the oxidation of cellular membrane lipids, and slow lipid peroxidation and microbial contamination of highly perishable fresh foods, such as chilled mackerel and chicken burgers, hence preserving their nutritional and microbiological quality and freshness [12-14].

The phenoxazinone ring forms the core structure of certain antibiotics produced from *Streptomyces antibioticus* and the fungus *Pycnoporus cinnabarinus*, while also being present in the ommochrome pigments of the eyes and skin. The chromopeptide antibiotic actinomycin D is commonly used in the treatment of a wide range of cancers. It is also considered to contribute to the activity of phenoxazinone antibiotics, allowing the compounds to intercalate nucleic acids between specific base pairs (5´-GC-3´). The 2-amino group is involved in hydrogen bonding to the cytosine C5 residue, and therefore plays a key part in successful intercalation, which is why so many phenoxazinones are effective anticancer agents. Phenoxazinones have also been biosynthesized (using bovine haemoglobin) or chemically synthesized and were shown to have anticancer or cytotoxic activity, as well as vasorelaxing and antibacterial effects [15-17].

The solvent most commonly used to extract ommochromes is methanol acidified with 0.5-5% hydrochloric acid (MeOH/HCl [8, 18-20]; more recent work has used a combination of ethanol and acetic acid (CH_3_COOH) to isolate ommochrome pigments from a giant squid's skin (*Dosidicus gigas*) [12]; however, the color change associated with the redox potential of the solvents used has been poorly studied. In this work, we evaluated the effect of two types of solvent extraction, varying in redox capacity, on the types of ommochrome extracted, and also assessed the antimicrobial activity and oxidative stress induction mechanisms observed when foodborne pathogens of clinical interest are exposed to ommochrome pigments from the skin of *Octopus vulgaris*.

## Materials and Methods

### Materials

Octopus (*Octopus vulgaris*) specimens were caught off the coast of Bahía de Kino (Sonora, Mexico; 28° 49′ 00 ′′N 111° 56′ 00′′ W, 15-18°C) in November 2018 and January 2019. All the samples were transported in plastic boxes with ice to the Marine Laboratory of the Food Research and Postgraduate Department (DIPA) of the University of Sonora and kept frozen at -20°C for later use.

### Isolation of Chromatophoric Granules and Pigment Extraction

The octopus samples were thawed at room temperature and the epidermal and dermal tissue contained in the chromatophore sacs were manually removed with the help of scissors; they were then lyophilized at -46°C (Labconco, USA) for 5 days. The lyophilized skin was pulverized in a grinder (Krups, Germany) and stored at 4°C while protected from light until the extraction process. The isolation of the chromatophoric granules and the extraction of the pigments (ommochromes) were carried out by means of two processes of solubilization of the ommochromes in acidified methanol (1% v/v HCl or 1% v/v acetic acid), followed by successive steps of centrifugation and sonication.

To do this, 1 mg of lyophilized skin was added to 20 ml of the methanol-HCl or methanol-acetic acid solution, respectively. The mixture was kept under constant stirring for 10 min (Vortex, USA) and sonicated in an ultrasonic bath (Branson BNS-M-1800, USA) for 10 min at room temperature 25°C. The solution was centrifuged at 2,000 *×*g at 4°C for 30 min (Sigma, USA) and the pigmented supernatant was collected. The extraction was repeated 3-4 times until no more color was collected. Finally, the pigmented supernatants were concentrated on a rotary evaporator (R-210, Buchi, Switzerland) at 37°C [8, 14].

### Structural Characterization and Antioxidant Activity of Ommochrome Pigments

The ommochromes obtained with each type of solvent, methanol-HCl and methanol-acetic acid, were characterized by ultraviolet-visible (UV-vis) spectrophotometric measurements carried out on a Cary 60 double beam spectrophotometer in a range of 200 to 800 nm with 10.0 nm interval. The blank solution was 1% (v/v) methanol-HCl and 1% (v/v) methanol-acetic, respectively, for each of the ommochromic extracts. In addition, Fourier-transform infrared (FTIR) spectroscopy was carried out using a Perkin Elmer FTIR GX spectrometer USA) by applying 16 scans in the 4,000–400 cm^-1^ spectral range. Spectra were measured using samples in KBr pellets.

### Microbial Strains and Inoculum Preparation

For the antibacterial evaluation, the gram-negative pathogenic bacteria *Escherichia coli* (ATCC 25922), *Salmonella* Typhimurium (ATCC 14028), *Pseudomonas aeruginosa* (ATCC 9027), *Pseudomonas fluorescens* (ATCC 13525) and gram-positive bacteria *Staphylococcus aureus* (ATCC 6538) and *Bacillus subtilis* (ATCC 6633), were used. Culture growth on nutrient agar at -20°C was transferred to plates (Difco Laboratories, USA) and incubated at a temperature of 37 ± 1°C for two days.

The fungi evaluated, which are considered among the most economically significant and clinically important pathogens in plants, were *Candida albicans* (ATCC 10231), *Aspergillus parasiticus* Spear, anamorph (ATCC 16992), *Alternaria* spp. (tomato isolate) and *Fusarium verticillioides* (Sacc) Nirenberg (synonym *Fusarium moniliforme* J. Sheld, anamorph (ATCC 52539). In antifungal assays all cultures were maintained on potato dextrose PDA Mueller-Hinton agar (Difco Laboratories) refrigerated at 4°C.

Bacterial inocula were prepared from one colony of each bacterial strain transferred to 5 ml of nutrient broth and incubated for 24 h at 37°C (Binder, USA). The bacterial cultures were diluted with nutrient broth for adjustment to 0.5-1.0 McFarland scale (1-3 × 10^8^ bacteria/ml) or to an OD at ~0.1 (600 nm). To obtain the fungal inocula, a spore suspension of each of the fungal cultures was prepared with 20 ml of sterile 0.1 % (v/v) Tween 80 solutions with constant stirring by means of a magnetic bar, for 10 min. The spore suspensions were adjusted to a concentration of 2 × 10^6^ spores/ml by counting in the Neubauer chamber using a light microscope (Model CX311RTSF, Olympus, Japan).

### Determination of Antibacterial and Antifungal Activity Using Microdilution Assay

For the antimicrobial evaluation, the broth microdilution method was used [21]. The ommochrome extract collected with methanol-HCl was prepared at a stock concentration of 400 mg/ml in DMSO, from which serial dilutions were made in the medium in a concentration gradient of 0.31-10 mg/ml. To a 96-well plate were added 100 μl of the different concentrations of the ommochromes, 100 μl microorganism control, 100 μl solvent negative control (DMSO) in medium to ensure no influence of the solvent on microbial growth, 100 μl of chloramphenicol (50 or 100 μg/ml as positive antibacterial controls) or 100 μl of Amphotericin B or Flutriafol (100 μg/ml as positive antifungal controls), 100 μl medium control, and finally, 10 μl of the bacteria inoculum or spore suspension was added as appropriate.

The plates with bacteria were incubated at 37°C for 24 h, while the plates with fungi were incubated at 30°C for 24, 48, and 72 h. The median inhibitory concentration (IC_50_) was calculated as the concentration that halved cell growth compared to the control. At the end of the incubation period, to the plates treated with the ommochromes as an antimicrobial inhibitor were added 10 μl of (3- (4,5-dimethylthiazol-2-yl) -2,5-diphenyltetrazolium (MTT) to the growth indicator at concentration of 5 mg/ml or 20 μl of 0.02% resazurin in water.

### Cell Viability Assay by MTT and Resazurin

To evaluate the cellular growth of the bacteria and fungi treated with the ommochromes, the method of reducing the tetrazolium salt MTT was used. The MTT metabolic reduction method is based on the reduction of 3- (4.5-dimeltiltiazol-2-yl) -2.5-diphenyltetrazolio bromide (MTT). This pale yellow, water-soluble dye is reduced early in viable cells by components of the respiratory chain (mitochondrial dehydrogenases) to formazan (blue-violet crystals, insoluble in water). Absorbance was measured at a wavelength of 570nm in a spectrophotometer (Thermo Scientific Multiskan Spectrum) [22].

The other quantitative colorimetric method used for determining cell proliferation capacity was resazurin (Alamar blue (7-hydroxy-10-oxidophenoxazin-10-io-3-one) non-fluorescent blue). Resazurin is reduced to resorufin (pink, highly fluorescent) by oxidoreductases that are found mainly in the mitochondria of viable cells. The absorbance is measured at a wavelength of 570 nm and/or fluorescence at 530 nmex and 590 nmem, since this dye has both chromophore and fluorophore properties [23].

### Live/Dead Cellular Double Staining Kit

The double staining kit was used to measure the simultaneous fluorescence of viable cells and dead cells. Viable cells are stained with calcein acetoxymethyl (Calcein AM), a non-fluorescent molecule that permeates the intact cell membrane. Inside the cell, the molecule is cleaved by intracellular esterases to yield calcein with an intense fluorescent green color. The double staining was performed with propidium iodide (KI), a non-fluorescent molecule that is impervious to the intact plasma membrane. KI can be intercalated to cellular DNA and emits a red fluorescence, indicative of the disintegrated membranes of dead cells.

To carry out the cell labeling, the procedure protocol was followed (Sigma-Aldrich 04511). The working fluorescence reagent was prepared on the spot by adding 10 μl of calcein-AM and 5 μl of KI to 5 ml of PBS. At the end of the time of treatment with ommochromes, 50 μl of the fluorescence reagent was added to the incubated plates, and they were kept between 15-30 min at 37°C; finally, the fluorescence was detected using a fluorescence microscope (Model CX311RTSF, Olympus).

### Intracellular Reactive Oxygen Species (ROS) Kit

The ROS Intracellular Fluorometric Kit especially detects superoxide and hydroxyl radicals in living cells. ROS react with the fluorogenic sensor (2,7 dichlorofluorescein diacetate (DCFH2-DA) resulting in a green fluorescent product. The assay was carried out following the manufacturer's recommendations (Sigma-Aldrich MAK 143). Twenty microliters of ROS detection reagent reconstituted and frozen at -20°C was added to 10 ml of the test buffer to form the fluorescence reaction solution (stable for 2 h). To the incubated plates, 50 μl of the fluorescence solution was added; they were kept 1 h at 37°C, and finally the fluorescence was detected using a fluorescence microscope.

### Fluorescence Microscopy

Signals emitted in the Live/Dead Kit (Sigma-Aldrich 04511) and ROS Kit (Sigma-Aldrich MAK 143) assays used to examine the mechanisms of cell death and proliferation were analyzed in an inverted epifluorescence microscope (Model DMi8 , Leica Microsystems, Germany) equipped with fluorescence filters (546/10 RHOD excitation filter and 585/40 emission, DAPI 350/50 excitation filter and 460/40 emission, and FITC excitation filter 480/40 and emission 527/30), a monochrome DFC 450C camera (Leica Microsystems) and fluorescence overlay software (LAS AF ver. 3.1.0, Leica Microsystems CMS GmbH). Since the ommochromes are capable of absorbing ultraviolet rays and can interact with ultraviolet light, the fluorescence emission in the suspension of methanol/HCl pigments (0.31-10 mg/ml) in nutrient broth medium (pH 7.4) was evaluated.

### Statistical Analyses

Descriptive analyses using FTIR, NMR and fluorescence microscopy were performed. Data were expressed as the mean ± SD. The IC_50_ was calculated through a regression analysis. Significance between experimental groups was determined by GLM-ANOVA followed by post hoc Tukey using NCSS software. Values of *p* ≤ 0.05 were considered statistically significant. Figures were created using Origin 8.0 software, and the error bars represent the SD.

## Results

### Chemical Structure Characterization

**Fig. 1:**
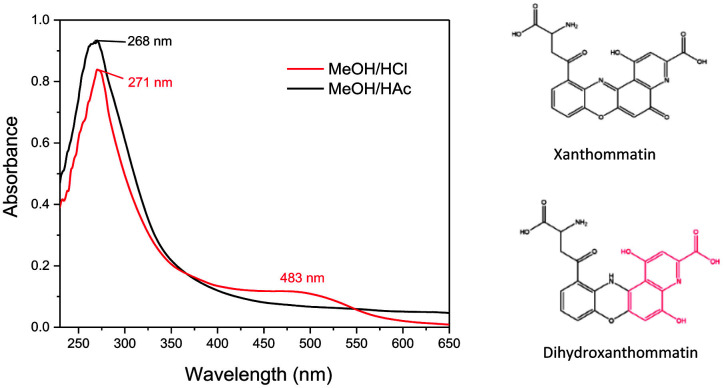
UV-Vis spectra of pigment extracts from the skin of *O. vulgaris* in methanol acidified HCl (Dihydroxanthommatin) or acetic acid (Xanthommatin).

Octopus ommochromes extracted from the chromatophore granules with acidified methanol (methanol-HCl or methanol-acetic acid) were characterized using UV-visible spectrophotometry. In the UV-visible region, characteristic peaks at 256, 367, and 400-550 nm were identified by their absorbance spectra in all ommochromes. The pH of the acidified methanol used for extraction was shown to influence the color of the extracted ommochromes. The typical peaks of different ommochromes can occur in the visible region in the 430-520 nm range. Extraction of the phenoxazinone-based ommochromes with MeOH/HCl (pH≈1) yielded pigments in a reduced state (oxo-pyrido[3,2-a]hydroxyphenoxazine),with a maximum absorbance at λ_max_ 483 nm; in contrast, yellow oxidized ommochromes (oxo-pyrido[3,2-a]phenoxazinone) λ_max_ disappeared in MeOH/acetic acid extraction (pH ≈ 6) (Fig. 1). In the UV region, the absorbance maxima at 268 and 271 nm are characteristic peaks of phenoxazinone extracted in acidified methanol.

This shows the influence of the proton transfer on the color and the electronic conjugation of chromophores. These results coincide with the UV-visible spectra characterized for these compounds. The color changes observed under reductive and oxidative conditions in the different acidified methanol solvents showed UV absorbance maxima (λ_max_) in the range characteristic of the phenoxazone and phenothiazine rings of the ommochrome (230-240 nm) [8, 24, 25]. Also, a similar yellow ommochrome compound, with a UV spectrum showing an absorption peak at 422 nm, was extracted from the giant squid in ethanol-acetic acid [12].

**Fig. 2:**
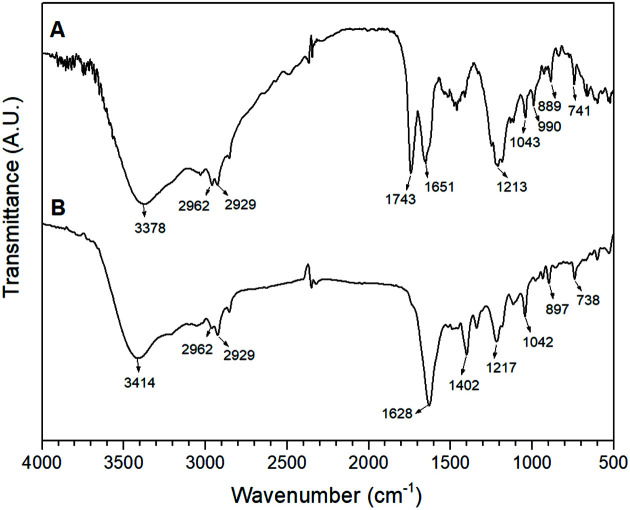
Infrared spectrum (FTIR) of pigments extracted in methanol-acids: [A] MeOH/HCl (1%v/v) and [B] MeOH/acetic acid (1% v/v).

One of the most important methods for identifying and characterizing ommochrome structure is Fourier-transform infrared spectroscopy (FTIR). The chemical structure of the octopus skin chromophores, as altered by methanol-acid extraction processes, was determined using this analysis. These spectra enable structural characterization of ommochromes by pointing to the presence of certain functional groups in the compounds. An increase in the intensity of characteristic chemical groups of the ommochromes was observed, influenced by the type of solvent used in the extraction. As seen in Fig. 2, the strong broad profile of the bands suggested the contribution of various vibrational modes in which aromatic rings and the characteristic OH and N-H groups of the unique primary amine in xanthommatin contribute to band A (3,018 to 3,281 cm^− 1^). The doublet of band B at 1,644 and 1,709 cm^-1^ indicates the N-H scissor mode, associated with a weak vibration mode of the aromatic ketone groups (C=O) that extended towards 1,768 cm^-1^. Band C reflects the C=N of the secondary amine of the pyridol ring at 1,152−1,344 cm^− 1^. Finally, band D includes characteristic vibrational modes of C−N and C−O aromatic stretching, as well as C−C aromatic stretching in xanthommatin [9, 12].

### Fluorescence Microscopy in Antimicrobial Activity

A cell viability Live/Dead double staining kit assay was performed to examine the potential cytotoxic effects of ommochromes on microorganisms. The assay marks live and dead cells, reflecting the permeability of cellular membranes. When ommochromes exert a cytotoxic effect that compromises the integrity of cell membranes, KI enters the cell and binds to its nucleic acids, resulting in a bright fluorescent red associated with cell death.

**Fig. 3:**
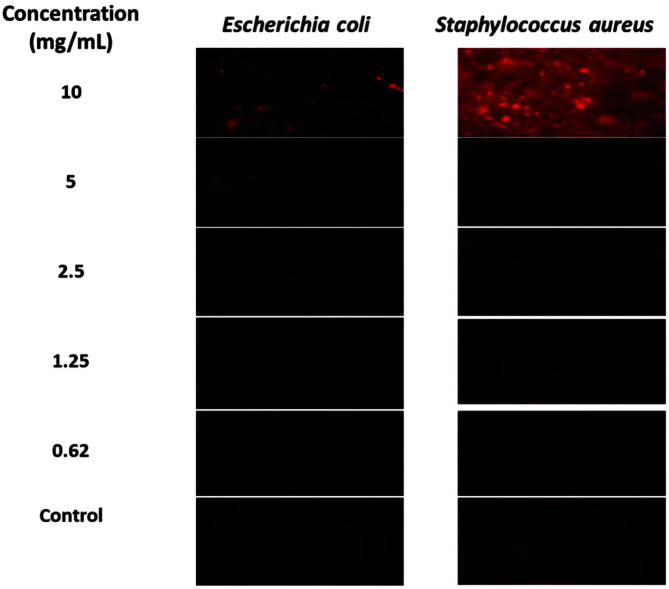
Epifluorescence (10x) images of *E. coli* and *S. aureus* stained with calcein-AM and propidium iodide (red fluorescence) Live/Dead Kit under treatment with ommochromes (10-0.62 mg/ml).

The fluorescence microscopy analyses were carried out on *E. coli* and *S. aureus*; these showed a high sensitivity to octopus pigments for both gram-negative and gram-positive bacteria, respectively. Immediately after the incubation time, the plates were treated with fluorochromes and the images were captured using the 10x objective (Fig. 3). The bacterial cultures treated with 10 mg/ml of ommochrome pigment showed an intense red fluorescence indicative of damage to the plasma membrane, in concordance with the > 80% of cell death as quantified by the MTT method. As might be expected, this fluorescence decreases as ommochrome concentrations are reduced, in agreement with the dose-response effect observed in the MTT reduction assay.

**Fig. 4:**
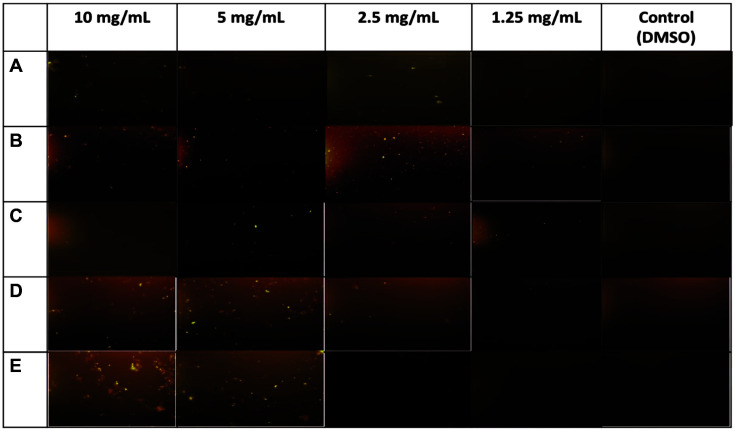
Epifluorescence images (at 10x) of (**A**) *S. aureus*, (**B**) *B. subtilis*, (**C**) *E. coli*, (**D**) *S. Typhimurium*, and (**E**) *C. albicans* treated with ommochromes and stained with fluorochrome from ROS Kit.

Induction of ROS (reactive oxygen species) associated with oxidative stress can be a highly effective antimicrobial mechanism [26]. Therefore, in this work the ability of ommochromes to disturb the plasma membrane and generate ROS was evaluated as a potential cytotoxic mechanism when microbes are grown in media containing ommochromes (10-1.25 mg/ml). Intense fluorescent signals were observed (Fig. 4), which increased as the concentration of the pigments increased. This fluorescence signal may reflect generation of ROS; however, it is important to consider that ommochromes have the ability to interact with UV-visible light during the visual cycle in the eyes of invertebrates, and therefore may influence the fluorescence signal.

**Fig. 5:**
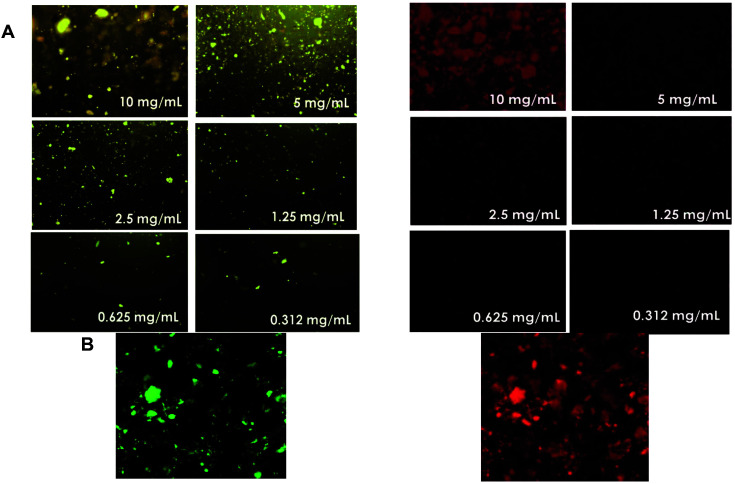
Intrinsic fluorescence images of octopus ommochromes at concentrations of 10-0.31 mg/ml in nutrient broth medium. On the left (blue filter) and on the right (green filter), using (**A**) epifluorescence microscopy and (**B**) Confocal scanning laser microscopy, both taken from the ommochrome extract with a concentration of 10 mg/ml ommochromes.

Taking the above into account, and considering that the intensity of the fluorescence signal was directly related to the amount of ommochromes present in the medium, we observed the interference of these pigments in the fluorescent signals emitted in the different filters of the microscope. Contrary to expectations, we found that the ommochrome pigments showed intrinsic fluorescence, which may be determined by the inherent chemical structure of these compounds (Fig. 5). The fluorescence signals increased with increasing concentration of the ommochrome pigment. Due to detection of autofluorescence, this assay may not be robust, and therefore other tests were conducted. However, the autofluorescence was unexpected, and the result is presented to the reader.

Given the interference of the ommochromes with the intrinsic fluorescence, two alternative methodological strategies were established to eliminate the interference signals. The first method consisted of performing two gentle washes of the wells with PBS and replacing the content of the wells with fresh medium, followed by the addition of the double-stained fluorochromes, with calcein-AM and propidium iodide; the second method was to filter the ommochrome solution through a 0.22 μm Millipore filter. This filtration process was not used due to the high retention of ommochrome pigments in the filter.

**Fig. 6:**
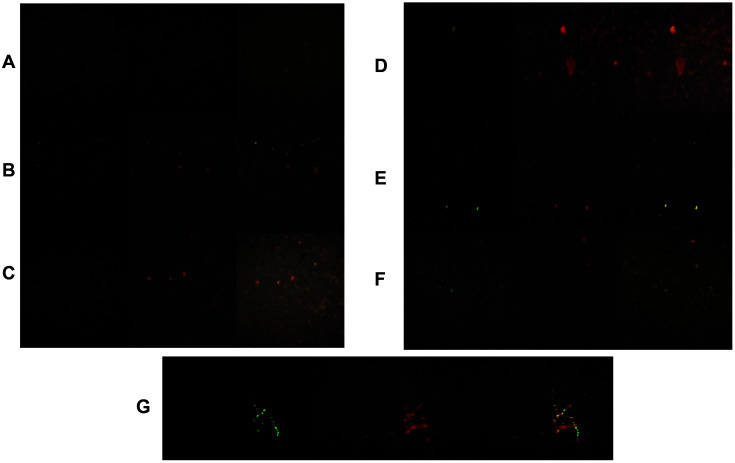
Microphotographs (10X) obtained in a confocal microscope of microorganisms treated with ommochromes (10 mg/ml) and stained with fluorochrome calcein-modified (row 1) and propidium iodide (row 2) and overlapping both fluorophores (row 3). (**A**) *S. aureus*, (**B**) *B. subtilis*, (**C**) *E. coli*, (**D**) *S. Typhimurium*, (**E**) *P. aeruginosa*, (**F**) *P. fluorescens*, and (G) *C. albicans*.

In addition to fluorescence microscope, scanning laser confocal microscopy (Fig. 6) was used to observe the effect after eliminating the ommochromes’ interference on the viability of microbial cells stained with fluorescent markers. For treatment at the ommochrome concentration of 10 mg/ml with extra washing, a moderate inhibitory effect was observed, due to the high intensity of the fluorescence of living cells (green fluorescence) in relation to the signal of dead cells (red fluorescence). This effect is more evident when overlapping both fluorophores for *S. aureus*, *B. subtilis*, *E. coli*, *S. Typhimurium*, *P. aeruginosa*, *P. fluorescens*, and *C. albicans*.

### Antibacterial Activity Assay

**Fig. 7:**
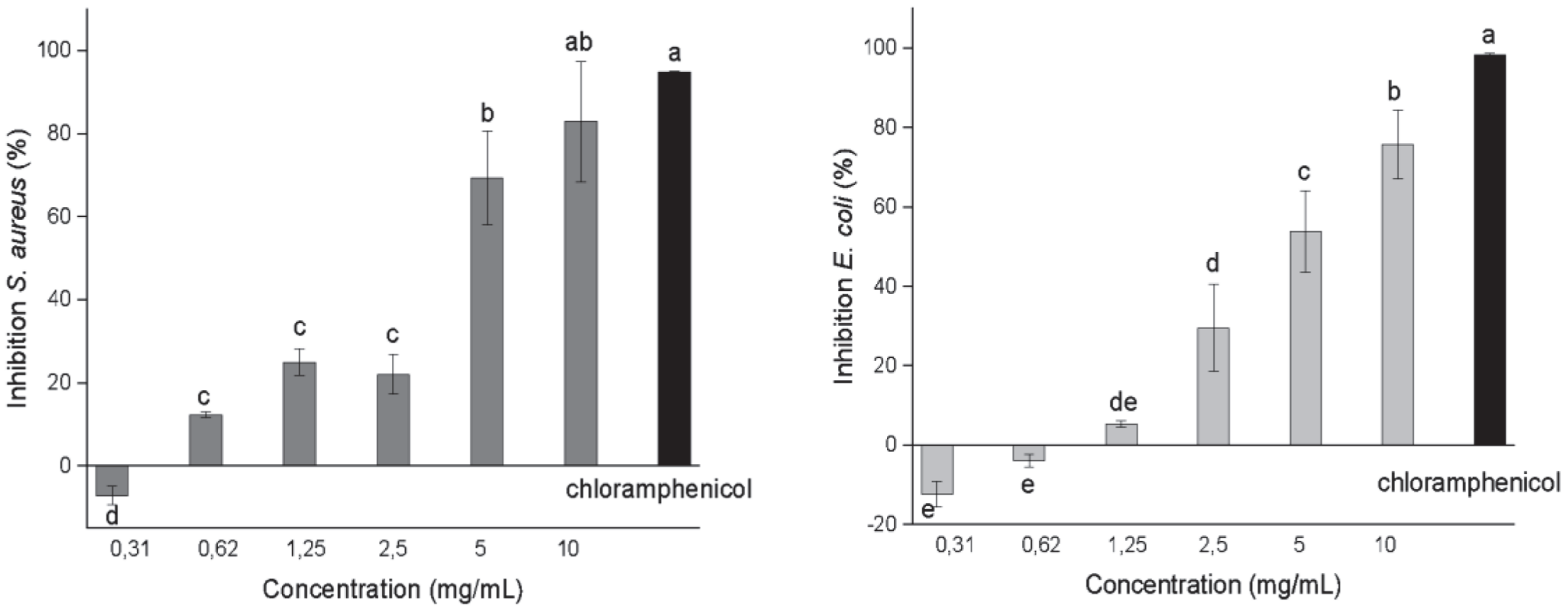
Cellular turbidity inhibition (OD_600_) after 24 h of treatment with ommochromes at concentrations of 0.31 to 10 mg/ml against *S. aureus* and *E. coli* using chloramphenicol (100 μg/ml) as a positive control. Results represent the mean ± SD of at least 3 experiments, in triplicate. The different letters indicate significant differences (*p* < 0.05) in each microorganism.

The various cell viability methods used to determine the inhibitory effects of the ommochrome isolates showed differences (*p* ≤ 0.05) in antibacterial activity. The cytotoxicity of the ommochromes against *S. aureus* and *E. coli* was evaluated with optical density at a λ of 600 nm. It was found that ommochromes have a concentration-dependent inhibitory effect, reaching up to 90% inhibition at a concentration of 10 mg/ml at 24 h (Fig. 7); a concentration much higher than that in which chloramphenicol acts as an antibiotic (100 μg/ml), was used as a positive control. The mean inhibitory concentration (IC_50_) was 5.13 and 5.07 mg/ml for *S. aureus* and *E. coli*, respectively, and a slight sensitivity (*p* > 0.05) was observed for *E. coli* compared to *S. aureus*. To corroborate this result, the determination of biological activity was carried out using colorimetric spectrophotometric methods.

From the absorbance values o f the formazan reduction (MTT) by viable cells, the percentages of cellular inhibition were calculated after a treatment period of 24 h of exposure, yielding findings similar to those previously described.

**Fig. 8:**
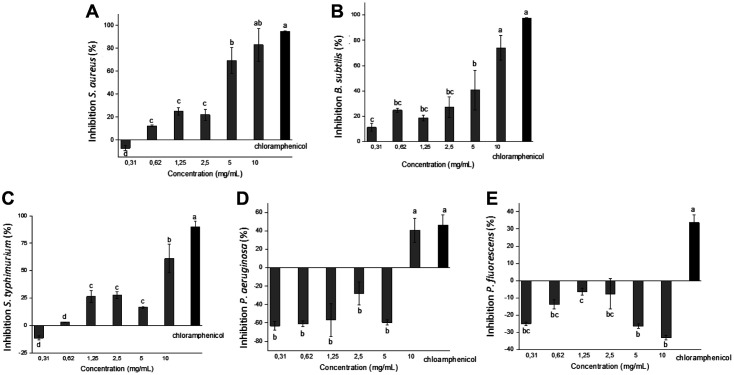
Cellular inhibition by the MTT reduction method after 24 h of treatment with ommochromes at concentrations of 0.31-10 mg/ml, for (**A**) *S. aureus*, (**B**) *B. subtilis*, (**C**) *S. typhimurium*, (**D**) *P. aeruginosa*, and (**E**) *P. fluorescens*, using chloramphenicol (100 μg/ml) as a positive control. The results represent the mean ± standard deviation of at least 3 independent experiments in triplicate. Different letters indicate significant differences (*p* < 0.05).

The ommochrome pigments showed significant differences (*p* ≤ 0.05) between the bacterial species. For *S. aureus* and *B. subtilis*, the survival values obtained corroborated the concentration-dependent effect of ommochromes; at 10 mg/ml, an important antibacterial effect is achieved, with inhibitions ~ 80%, demonstrating reproducible results. The IC_50_ values were 5.1 and 6.18 mg/ml for *S. aureus* and *B. subtilis*, respectively, comparable to the results of the average inhibitory concentrations obtained using turbidimetry assay. Likewise, the chloramphenicol (100 μg/ml) showed a 90% inhibition for gram-positive bacteria (Fig. 8).

When evaluating the cytotoxicity of the ommochrome pigments on *S. Typhimurium*, *P. aeruginosa*, and *P. fluorescens* using the MTT method (Fig. 8), a lower sensitivity (*p* ≤ 0.05) was obtained for this group. However, *S. Typhimurium* showed a concentration-dependent toxic effect with an IC_50_ of 7.25 mg/ml, slightly lower than the inhibitory effect obtained for gram-positive bacteria. Contrary to expectations, pigments did not decrease cell viability for *Pseudomonas* species. In *P. fluorescens*, the proliferation percentages exceeded 100, showing a high resistance to antimicrobial action for this species. Only at the maximum concentration (10 mg/ml) was a significant reduction of ~50% observed for *P. aeruginosa*; no effect was observed at lower concentrations. However, it is noteworthy that, in the chloramphenicol treatment, the inhibition percentages only reached between 30-50%, indicative of the high resistance of this group of bacteria.

The cellular reduction of resazurin to resorufin, yielding a highly fluorescent pink color, is catalyzed by oxido-reductases present in the mitochondria of living cells, and hence is indicative of cellular viability. This method can quickly detect the sensitivity of bacteria and yeasts to ommochromes, as viable cells become a fluorescent pink, whereas non-viable cells remain blue. To quantify the inhibitory effect of the ommochromes, the intensity of the fluorescence of resorufin was measured.

**Fig. 9:**
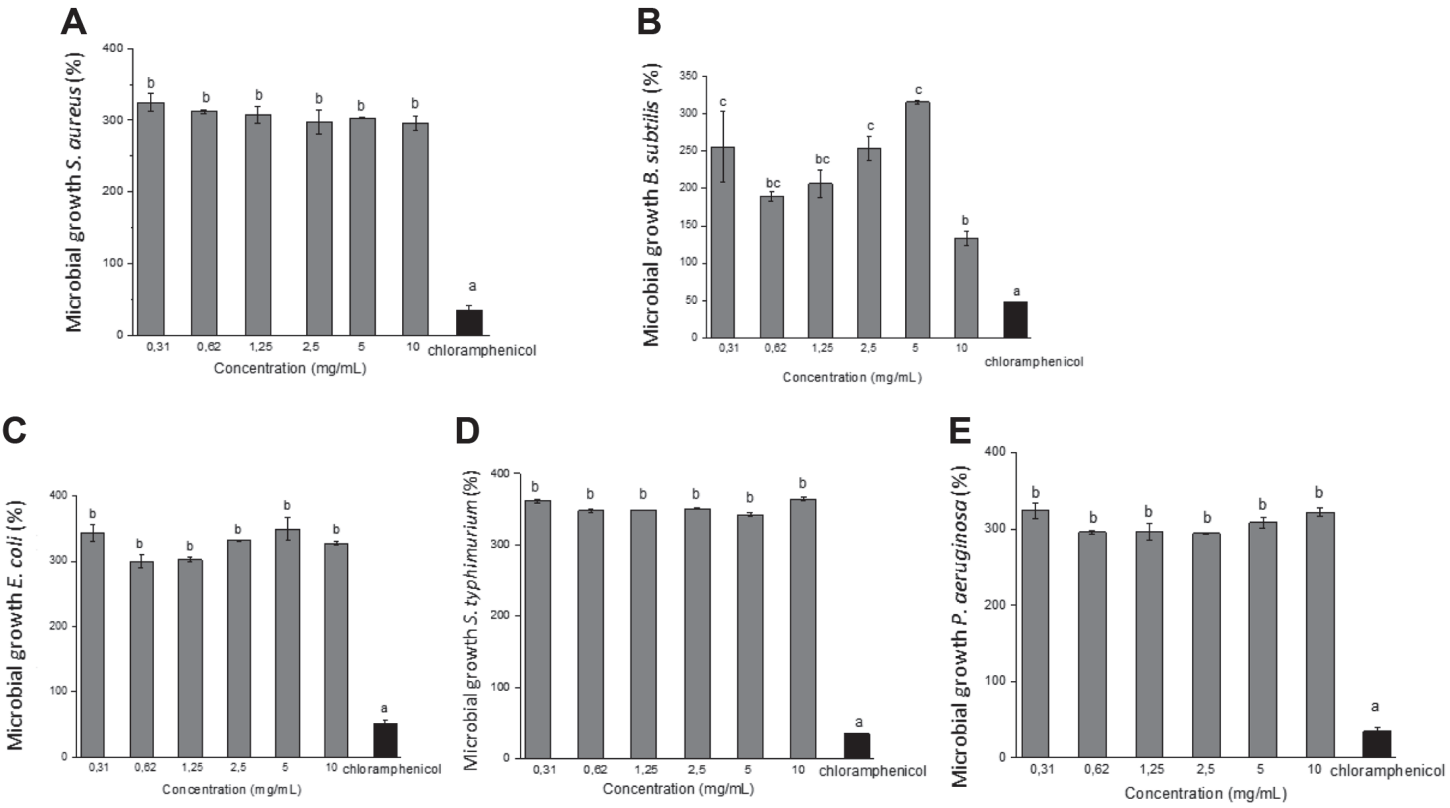
Microbial growth (%) of bacteria treated with ommochromes (0.31-10 mg/ml) at 24 h, determined by the resazurin method for (**A**) *S. aureus*, (**B**) *B. subtilis*, (**C**) *E. coli*, (**D**) *S. Typhimurium*, and (**E**) *P. aeruginosa*, using chloramphenicol (100 μg/ml) as a positive control. Results represent the mean ± standard deviation of at least 3 experiments in triplicate. The different letters indicate significant differences (*p* < 0.05).

Cell growth was increased in samples treated with different concentrations of ommochromes (10-0.31 mg/ml). The viability values o btained by this method ranged up to ~ 300% of cell proliferation (Fig. 9), showing that at the concentrations used, no cytotoxic effect (*p* > 0.05) was seen (indeed, growth increased) on the different bacterial strains. This suggests that ommochrome pigments have a low inhibitory effect.

To rule out any reduction interference given by a redox change of the ommochromes themselves, we verified that the pigments did not induce the color change. We then established that the intensity of the fluorescent pink color of the visual detection is caused by microbial metabolism itself. This response is consistent with that obtained by visual detection of color change. For *B. subtilis* we observed that the microbial growth increased proportionally with the concentrations of ommochrome pigments, with the exception of the maximum concentration (10 mg/ml). On the other hand, the use of chloramphenicol at a concentration of 100 μg/ml exerted a cytotoxic effect, reducing viability by over 50%. However, staining methods differ markedly between MTT cell proliferation responses and with resazurin.

### Antifungal Activity Assay

Although antifungal compounds have had a lower potential for development than antibacterial substances, there is currently an increasing interest in naturally-occurring antifungal agents that can decrease their toxic or adverse effects, and minimize their resistance to conventional formulations. In this regard, an important microbial group of interest are filamentous fungi, which are involved in clinically important food-borne diseases. The antifungal activity of the ommochromes was evaluated against the yeast *C. albicans* and the filamentous fungi *A. parasiticus*, *Alternaria* spp., and *F. verticillioides*.

**Fig. 10:**
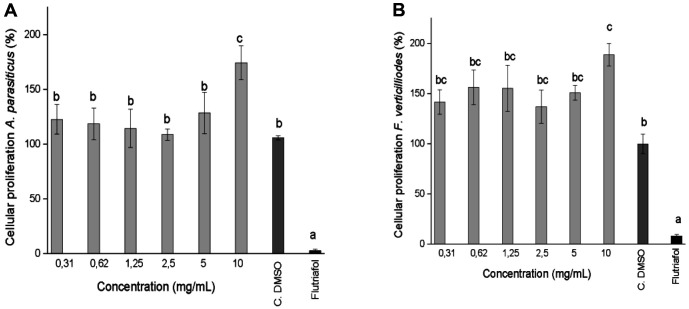
Cell growth (OD_600_) of *A. parasiticus* (**A**) and *F. verticillioides* (**B**) after exposure to the ommochromes (0.31 to 10.0 mg/ ml), Flutriafol (agricultural fungicide 100 μg/ml) and cell control with and without DMSO. The bars represent the mean ± SD, different letters indicate significant differences (*p* < 0.05).

Filamentous fungi *A. parasiticus* and *F. verticillioides* treated with ommochromes in a concentration gradient of 0.31 to 10.0 mg/ml at 24 h of incubation showed a null inhibitory effect measured by the turbidity of the well at 600 nm, compared with the control DMSO cell (100% viability). Meanwhile, the agricultural antifungal Flutriafol (100 μg/ml), used as a positive control, showed potent fungicidal power (viability < 2%) (Fig. 10).

The microscopic characteristics of the fungi observed at the different concentrations of ommochromes allow us to conclude that there are no alterations in the structures of the conidia, which keep their spherical or globose and curved or canoe-shaped forms for *A. parasiticus* and *F. verticillioides*, respectively. In addition, a large number of germinated spores and hyphae were found, which indicates that the fungi continue their metabolism and development in a normal way.

**Fig. 11:**
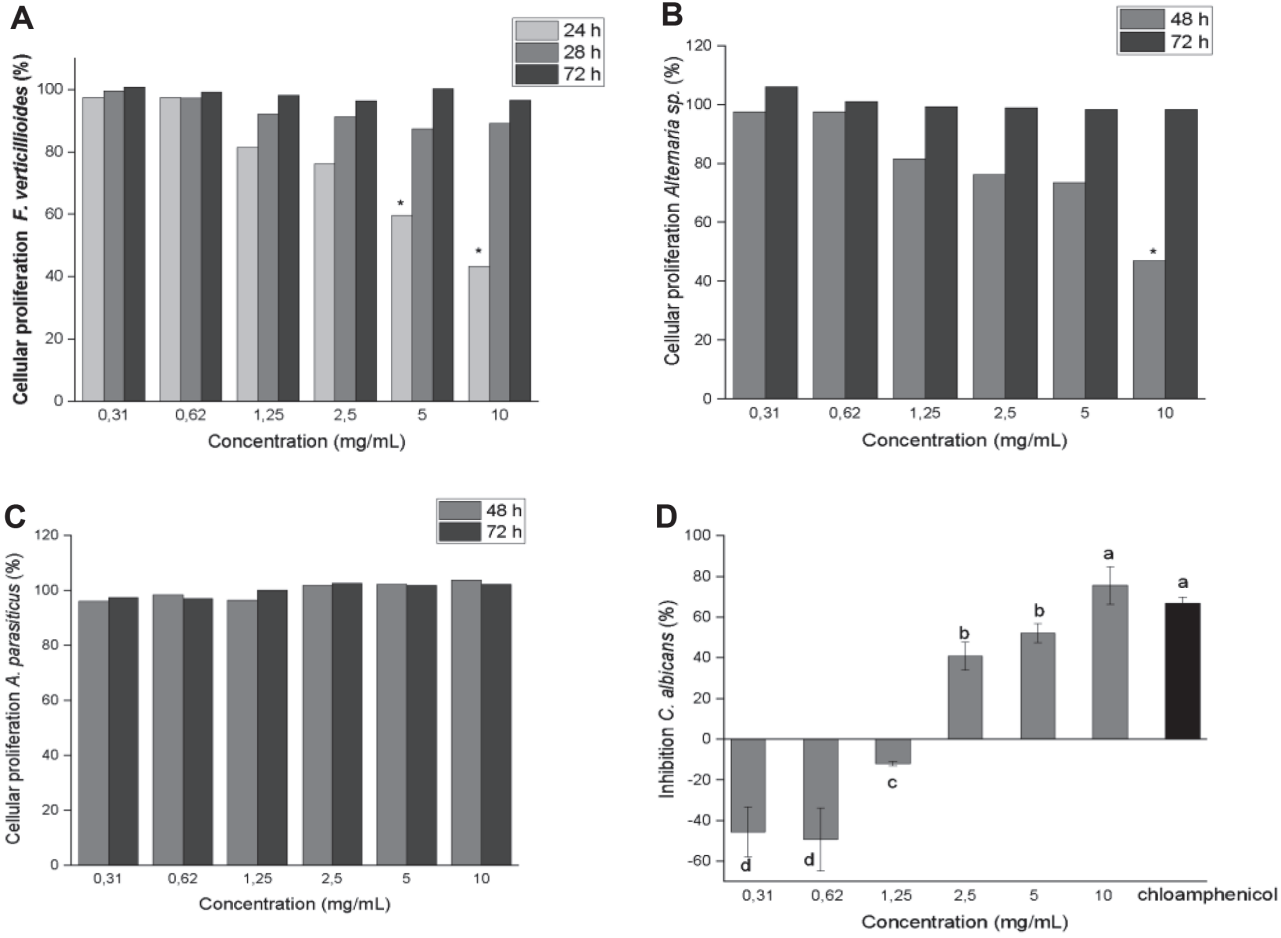
Cell viability of *F. verticillioides* (**A**), *Alternaria* spp. (**B**), *A. parasiticus* (**C**) and *C. albicans* (**D**), after exposure to the ommochrome extract at concentrations of 0.31 to 10.0 mg/ml, at 24, 48 and 72 h of incubation.

In parallel, the cell viability of *Alternaria* spp., *A. parasiticus*, *F. verticillioides*, and *C. albicans* treated with ommochromes (10.0 to 0.31 mg/ml) was evaluated using the tetrazolium MTT salt reduction assay for 24, 48, and 72 h (Fig. 11). As observed at 24 h, the metabolic rate of MTT reduction was low (*p* ≤0.05), with the exception of *F. verticillioides*, which showed a high (*p* ≤0.05) growth density with a concentration-dependent inhibitory profile of the ommochromes. At that time, at concentrations of 5 and 10 mg/ml, there was between 20 and 40% inhibition in fungal growth; however, this effect is lost at 48 and 72 h. This result is consistent with the morphological alterations observed in the fungal cells at an ommochrome concentration of 10.0 mg/ml, indicative of the antifungal effect of these compounds.

At 48 and 72 h, there was a marked difference in the ability to metabolize MTT by different fungi, while *F. verticillioides* and *Alternaria* spp. drastically reduced the tetrazolium salt. For *A. parasiticus* a lower metabolic activity was observed, which was more accentuated at 72 h, at which time the ommochromes had no inhibitory effect. It is shown that, in *Alternaria* spp. at 48 h, the maximum concentration (10 mg/ml) achieved an inhibition of 40%; however, this activity was not maintained at 72 h – an effect similar to the fungistatic behavior seen with *F. verticillioides*.

These results suggest that the action of the ommochromes is fundamentally fungistatic, unlike Flutriafol (100 μg/ml), whose strong fungicidal effect remains until 72 h after the experiment. Although *F. verticillioides* and *Alternaria* spp. showed some sensitivity to ommochromes, for *A. parasiticus* no inhibitory effect was observed at the concentrations and times evaluated, indicating greater resistance. In general, the growth of the different filamentous fungi was slightly inhibited by the ommochromes, the effect becoming minimal at 72 h, when the growth activity is comparable to that of cellular controls.

Unlike the filamentous fungi that showed greater resistance to the inhibitory action of the ommochrome pigments, the yeast *C. albicans* showed greater sensitivity (*p* ≤ 0.05) to these compounds at 24 h. A concentration-dependent inhibitory effect was observed, a significant inhibition of cell growth being detected with the 2.5 mg/mL concentration of ommochromes. For this microorganism, the mean inhibitory concentration value (IC_50_) was 5.99 mg/ml. The growth of the cells inoculated in the medium with the maximum concentration evaluated (10 mg/ml) was inhibited by 75%, comparable to the fungicidal effect observed with a hundred-fold lower concentration of the positive control chloramphenicol (100 μg/ml) (Fig. 11).

**Fig. 12:**
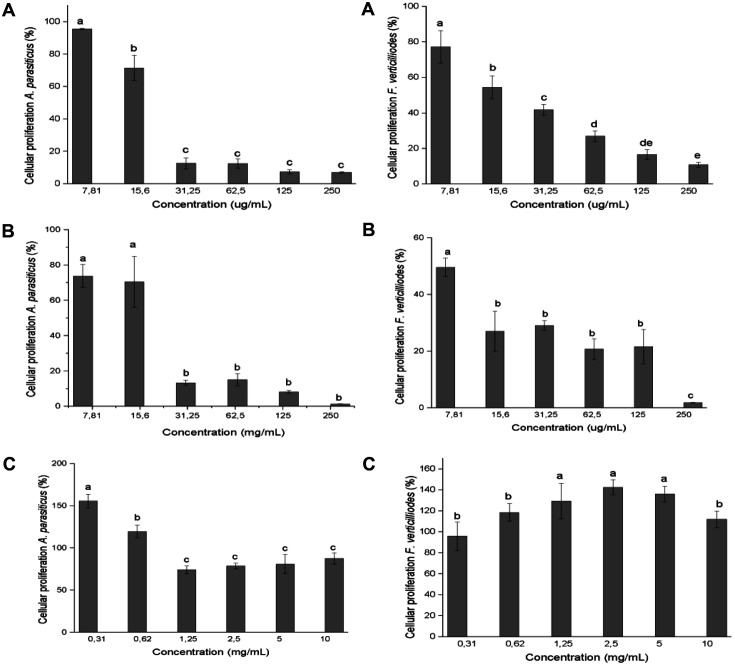
Antifungal effect of Flutriafol (**A**), Amphotericin B (**B**) at concentrations of 7.81-250 μg/ml, and Quinazo (**C**) (0.31-10.0 mg/ml) on the viability of spores of *A. parasiticus* and *F. verticillioides* at 24 h incubation. The bars represent the mean ± SD, different letters indicate significant differences (*p* < 0.05).

The agricultural antifungals Flutriafol and Quinazo (plant extracts), and Amphotericin B (commercial polyene antifungal) showed different inhibitory activities on *A. parasiticus* and *F. verticillioides* as shown in Fig. 12.

The synthetic compounds (Flutriafol and Amphotericin B) in a concentration gradient (7.81-250 μg/ml) had a strong concentration-dependent fungicidal effect. This toxic activity was more pronounced for *A. parasiticus* compared to *F. verticillioides*, a result divergent from that seen with ommochromes, in which *F. verticillioides* was more sensitive. When comparing the ommochrome pigments with the natural fungicide Quinazo in concentrations of 0.31-10.0 mg/ml, similar results were obtained with a low inhibitory effect on the evaluated fungi.

## Discussion

Natural phenoxazinone, which contain a tricyclic iminoquinone system, are present in ommochromes (xanthommatin and dihydro xanthommatin) and ommines, constituting the pigments responsible for the color of the skin and eyes of invertebrates. The solvent most used in the extraction of ommochromes is methanol acidified with 0.5-5% hydrochloric acid (MeOH-HCl). This allows the extraction of ommochrome precursors, most ommatins, and to some extent ommines. Only ommatin D and rhodomatine can be extracted with neutral aqueous solvents due to the presence of sulfate and glucose, respectively [5, 8, 27].

The ommochromes are commonly identified by their absorbance spectra with three characteristic peaks at 256, 367, and 463 nm [8, 20, 24]. Color reduction under reducing conditions (MeOH/HCl, pH≈ 1) may occur due to increased electronic delocalization of the phenoxazine chromophore (H_2_-xanthommatin), leading to further resonance stabilization of the oxonium ion (O^+^). The fact that the ommochrome pigments obtained with MeOH/HCl had twice the antioxidant activity may be due to their reduced structure that favors the extension of their electronic delocalization, in contrast with the pigments extracted in MeOH/ acetic acid. Since the anti-radical property of these compounds is directly related to the N-H bond, this implies that only the reduced ommochromes can act as potent anti-radicals and antioxidants. In the reduced state of these pigments, there is greater resonance stabilization, which decreases the enthalpy of dissociation of the NH (I) bond; therefore, an increase in entropy during electronic delocalization further decreases the energy barrier of the N-H link dissociation (II). Finally, electron donor side chains could reinforce electronic delocalization (III) [28].

Recent studies suggested that the redox status and anti-radiation capabilities enable phenoxazine compounds to decrease oxidative stress [28, 29]. In vivo, it was shown that phenoxazines and phenothiazines are potent inhibitors of autooxidation and ferroptosis (iron-dependent oxidative stress) owing to trapping of lipid radicals, thus breaking the propagation mechanism [18, 30]. Interestingly, this antiradical property was directly related to the N-H bond of the phenoxazine/phenothiazine rings [28].

Currently, ommochromes have engendered great scientific and technical interest in the food, pharmaceutical, biomedical, materials, and electronics sciences, among others. The antimicrobial activity of cephalopod ommochrome pigments, such as those from the giant squid (*Dosidicus gigas*), has been previously evaluated by Aubourg *et al*. [12], Chan-Higuera *et al*. [14], and Esparza-Espinoza *et al*. [2021, https://doi.org/10.1590/fst.18221]. In addition, similar biological activities of insect ommochromes (fly: *Hermetia illucens*) have been reported [10, 11].

The results obtained in this study from octopus (*Octopus vulgaris*) ommochromes are in agreement with results reported for fly ommochromes (1 mg/disc), which showed a null growth halo inhibition activity for *Bacillus coagulans*, *S. aureus*, *E. coli*; inhibitory activity was only observed against *B. subtilis*, although the pigments showed lower activity compared to the antibiotic (Oxacillin 10 μg/ml) [11]. Similarly, the antibacterial effect evaluated by means of the inhibition halo agar diffusion technique (1 mg/disc), was classified as trace or low (for *Bacillus cereus*, *Clostridium perfringens*, *Klebsiella pneumoniae*), and as moderate (for *S. aureus*, *Haemophilus influenzae*, and *L. monocytogenes*). The greatest discrepancy between this study and ours was obtained with S. enterica; they reported high sensitivity, whereas our study did not detect a marked inhibition for *S. Typhimurium* [14].

The phenoxazinone system has the potential to intercalate within DNA chains; this has been considered as a basis for cytotoxicity in the development of antibiotic and anticancer drugs. In this sense, the ommochrome pigments showed a weak or moderate inhibitory activity depending on the type of microorganism. Gram-negative bacteria *S. Typhimurium*, *P. aeruginosa*, and *P. fluorescens*, were less inhibited by these compounds; this may reflect the structure of their cell walls, which is rich in phospholipids, lipopolysaccharides, lipoproteins, surface proteins and peptidoglycan. This can result in differences in the penetration and retention of chemical compounds [31, 32].

Furthermore, our results agree with reports by Shimizu *et al*. [16], who showed that different synthetic phenoxazinones did not exert an antimicrobial effect against *E. coli*, *P. aeruginosa*, *S. Typhimurium*, *S. aureus*, *L. monocytogenes*, or tuberculous mycobacterial strains, although they showed considerable effect against non-tuberculous mycobacteria. According to the authors, the great sensitivity of mycobacteria to phenoxazinone could be related to the unique characteristics of mycobacterial cell wall structure: an inner layer of peptidoglycan and arabinogalactan, an intermediate layer of mycolate, and an outer layer of lipoglycans, free of polysaccharides, glycolipids and phospholipids.

Although the phenoxazinone system has been thought to enable intercalation in DNA chains with cytotoxic effect, the fact that ommochromes such as phenoxazinone have shown a moderate effect on the inhibition of microbial growth can be attributed to the fact that the single nucleus of phenoxazinone is incapable of inhibiting cell proliferation. In this regard, this polycyclic coplanar structure has been reported to require hydrogen bond acceptor/donor groups and additional π sites, such as double bond substituents or fused rings, to promote stacking interactions with DNA [17]. This can be decisive for enzymatic blockade and reading errors to occur during the replication processes.

Regarding antifungal activity, the ommochromes showed a greater inhibitory effect on the growth of *C. albicans* than on the filamentous fungi *Alternaria* spp., *A. parasiticus* and *F. verticillioides*, as assessed by the broth microdilution method. This broth dilution method is an important standard for determining the in vitro susceptibility of both yeast and filamentous fungi. The hypersensitivity of *C. albicans* compared to the evaluated filamentous fungi may reflect differences in their cell walls.

Works carried out by Chan-Higuera *et al*. [14] and Dontsov *et al*. [11] reported a greater inhibitory effect of ommochromes determined by agar diffusion on *C. albicans* and *C. glabrata* as compared to *Aspergillus* flavus, *Aspergillus versicolor*, *Penicillium chrysogenum* and *Penicillium griseofulvum*. These results are analogue to those obtained in our work. However, they differ in that the octopus ommochromes evaluated by the microdilution method only showed significant inhibitory activity on *C. albicans*, with fungistatic inhibition.

In yeast cells, the composition of the membrane is associated with activity/resistance to azoles. Deletion of the UPC2 gene and inactivation of the sterol desaturase enzyme involved in ergosterol synthesis in *Saccharomyces cerevisiae* and in *C. albicans* resulted in cells that were hypersensitive to fluconazole and ketoconazole [33, 34]. Another essential factor for the sensitivity of *C. albicans* is the alteration in the composition of membrane sphingolipid, which suppresses the activity of the drug efflux pump and consequently increases susceptibility to azole activity [30]. However, the resistance mechanisms for filamentous fungi such as *Aspergillus* spp. are different from those of *Candida* spp., owing to a decrease of the 14-α-sterol demethylase protein, the overexpression of efflux pumps, and an increase of the barrier to drugs [35].

The present investigation evaluates the impact that the redox potential of the methanol/hydrochloric acid versus methanol/acetic acid combination has on the extraction capacity of different kinds of solvent, as well as its effect on the antioxidant potential of the ommochrome pigments. The results show: i. the redox state alters the color of the ommochromes and is dependent on the pH in the extraction solvent; while acidic conditions (MeOH/HCl) isolated reduced red ommochromes, neutral conditions (MeOH/acetic acid) extracted oxidized yellow pigments; ii. ommochromes' natural fluorescence apparently did not differ between solvent extractions and showed a strong fluorescence; iii. the vibrational modes of the FTIR and NMR-^1^H spectra arise from the presence of xanthommatin-like compounds; iv. natural phenoxazinone (octopus ommochromes) was shown to be moderately toxic for the pathogens *S. aureus*, *B. subtilis*, *S. Typhimurium*, and *C. albicans*; v. the species *P. aeruginosa*, *P. fluorescens*, and *F. verticillioides* were tolerant to this pigment.
